# A connectivity model of the anatomic substrates underlying Gerstmann syndrome

**DOI:** 10.1093/braincomms/fcac140

**Published:** 2022-05-27

**Authors:** Qazi S. Shahab, Isabella M. Young, Nicholas B. Dadario, Onur Tanglay, Peter J. Nicholas, Yueh-Hsin Lin, R. Dineth Fonseka, Jacky T. Yeung, Michael Y. Bai, Charles Teo, Stephane Doyen, Michael E. Sughrue

**Affiliations:** School of Medicine, University of New South Wales, 2052 Sydney, Australia; Omniscient Neurotechnology, Sydney 2000, Australia; Rutgers Robert Wood Johnson Medical School, New Brunswick, NJ 08901, USA; Omniscient Neurotechnology, Sydney 2000, Australia; Omniscient Neurotechnology, Sydney 2000, Australia; Centre for Minimally Invasive Neurosurgery, Prince of Wales Private Hospital, Randwick 2031, Australia; Centre for Minimally Invasive Neurosurgery, Prince of Wales Private Hospital, Randwick 2031, Australia; Centre for Minimally Invasive Neurosurgery, Prince of Wales Private Hospital, Randwick 2031, Australia; Centre for Minimally Invasive Neurosurgery, Prince of Wales Private Hospital, Randwick 2031, Australia; Centre for Minimally Invasive Neurosurgery, Prince of Wales Private Hospital, Randwick 2031, Australia; Omniscient Neurotechnology, Sydney 2000, Australia; Omniscient Neurotechnology, Sydney 2000, Australia

**Keywords:** Gerstmann’s syndrome, connectivity, connectome, network

## Abstract

The Gerstmann syndrome is a constellation of neurological deficits that include agraphia, acalculia, left–right discrimination and finger agnosia. Despite a growing interest in this clinical phenomenon, there remains controversy regarding the specific neuroanatomic substrates involved. Advancements in data-driven, computational modelling provides an opportunity to create a unified cortical model with greater anatomic precision based on underlying structural and functional connectivity across complex cognitive domains. A literature search was conducted for healthy task-based functional MRI and PET studies for the four cognitive domains underlying Gerstmann’s tetrad using the electronic databases PubMed, Medline, and BrainMap Sleuth (2.4). Coordinate-based, meta-analytic software was utilized to gather relevant regions of interest from included studies to create an activation likelihood estimation (ALE) map for each cognitive domain. Machine-learning was used to match activated regions of the ALE to the corresponding parcel from the cortical parcellation scheme previously published under the Human Connectome Project (HCP). Diffusion spectrum imaging-based tractography was performed to determine the structural connectivity between relevant parcels in each domain on 51 healthy subjects from the HCP database. Ultimately 102 functional MRI studies met our inclusion criteria. A frontoparietal network was found to be involved in the four cognitive domains: calculation, writing, finger gnosis, and left–right orientation. There were three parcels in the left hemisphere, where the ALE of at least three cognitive domains were found to be overlapping, specifically the anterior intraparietal area, area 7 postcentral (7PC) and the medial intraparietal sulcus. These parcels surround the anteromedial portion of the intraparietal sulcus. Area 7PC was found to be involved in all four domains. These regions were extensively connected in the intraparietal sulcus, as well as with a number of surrounding large-scale brain networks involved in higher-order functions. We present a tractographic model of the four neural networks involved in the functions which are impaired in Gerstmann syndrome. We identified a ‘Gerstmann Core’ of extensively connected functional regions where at least three of the four networks overlap. These results provide clinically actionable and precise anatomic information which may help guide clinical translation in this region, such as during resective brain surgery in or near the intraparietal sulcus, and provides an empiric basis for future study.

## Teaser

Shahab *et al*. report a connectivity model of the neuroanatomic substrates which are likely impaired in Gerstmann’s Syndrome. A frontoparietal network converging on three specific parcellations in the anteromedial portion of the intraparietal sulcus was found to be involved in calculation, writing, finger gnosis and left–right orientation.

## Graphical Abstract

**Graphical abstract:**
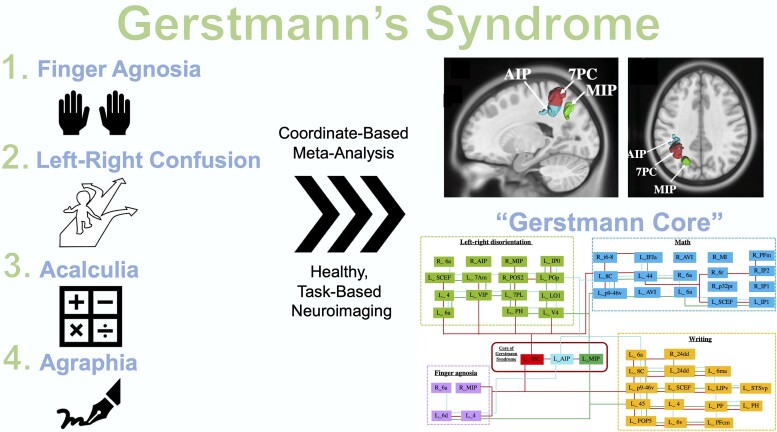

## Introduction

Gerstmann’s syndrome is a constellation of severe neurological deficits, namely agraphia, acalculia, finger agnosia, and left–right discrimination.^1,2^ It is generally agreed to occur with damage to the dominant parietal lobe, often following cerebral infarction. However, there is much debate regarding the exact location of a cortical lesion within the parietal lobe which can lead to such diverse clinical symptoms. This makes pure Gerstmann’s syndrome rare and very difficult to study.^3,4^

Since its first characterization in the 1920s, much attention has focused on the possibility of an underlying Grundstörung (common functional disturbance) in Gerstmann’s syndrome due to its diversity in symptomology amongst unique cognitive domains. Researchers and clinicians alike have both challenged and marvelled at the possibility of a united neural commonality amongst diverse neuro-behavioural functions.^5,6^ However, despite recent advancements in neuroimaging technology, few recent authors have since attempted to, nor been successful in, providing an accurate cortical model uniting Gerstmann’s functional impairments.^2,5,7^ Electrostimulation studies have proposed a spatial relationship based on proximity underlying the cognitive domains but have been unsuccessful in providing a unified model.^3,8^ Elsewhere, some have suggested the presence of common neural networks amongst functions of finger calculations and knowledge due to their similar methods of developmental acquisition in children, whereas others have disagreed with this notion and instead proposed shared networks only remain amongst functions of finger naming and left–right orientation.^1,6^ Importantly, Rusconi *et al*.^9,10^ more recently suggested Gerstmann’s syndrome may be related to intraparietal white matter disconnection following subcortical injury according to a study on a small sample of healthy subjects. While encouraging, the previous results have yet to be verified and could be further improved with coordinate-based methodology that can procure larger amounts of neuroimaging data with less bias across the literature.^9^ Furthermore, the white matter connectivity and large-scale brain networks underlying these neuro-behavioural domains in this region have yet to be previously explored in sufficient detail and may be addressed through the use of a more anatomically fine parcellation scheme for clinically actionable anatomic information.

In this context, we aimed to generate an anatomically-specific cortical model of the neuroanatomic substrates likely involved in Gerstmann’s syndrome. The challenges of previous work in creating a unified model of Gerstmann’s syndrome are likely related to the rarity of this disorder in addition to the heterogeneity of anatomical nomenclature utilized to compare results across the literature. Therefore, we employed a coordinate-based meta-analytic software to generate activation likelihood estimation (ALE) maps of previously reported task-based fMRI-PET studies on healthy subjects and then incorporated these results into a previously established and anatomically-specific parcellation scheme published under the Human Connectome Project (HCP).^11^ Through these functional analyses, we aimed to identify the most likely cortical regions which may contribute to the Gerstmann’s tetrad of symptoms. Furthermore, given the recent focus on a disconnection hypothesis of this clinical phenomenon, we sought to detail the connectivity of a possible Gerstmann Core network through region-based deterministic tractography specifically on key parcels identified in our functional analyses across each domain. The localization of neural substrates underlying the ability to write, perform mathematics, distinguish left from right, and discriminate one’s own finger to an anatomically parcellated network could not only help address the long-standing questions surrounding Gerstmann’s clinical phenomenon but also provides actionable anatomic information for clinical applications in this region, such as for resective brain surgery,^12^ modulatory treatments,^13^ or early prognostication.^14^

## Materials and methods

### Literature search

An extensive search strategy was devised and applied to the electronic databases BrainMap Sleuth 2.4 (http://brainmap.org/sleuth/) and Pubmed and Medline in three separate times between September 2018 and February 2019.^15–17^ Each database was queried for all years provided by the database since its inception up until 2019. Numerous search algorithms were utilized that focused on fMRI and PET studies in relation to the four cognitive domains (writing, mathematical calculations, finger gnosis, left–right discrimination) and their abnormal counterparts. The search strings included for our literature review are the follows:

1. ‘writing OR Agraphia OR Language AND fMRI’
2. ‘writing OR Agraphia OR Language AND PET’
3. ‘finger gnosis OR finger agnosia OR finger movement OR body structure representation OR motor attention OR finger sense OR finger configuration AND fMRI’
4. ‘finger gnosis OR finger agnosia OR finger movement OR body structure representation OR motor attention OR finger sense OR finger configuration AND PET’
5. ‘math OR calculation OR counting OR acalculia OR arithmetic OR numerical cognition AND fMRI’
6. ‘math OR calculation OR counting OR acalculia OR arithmetic OR numerical cognition AND PET’
7. ‘left–right discrimination OR visuospatial attention OR body knowledge OR body space relation OR body schema OR self-rotation OR mental rotation AND fMRI’
8. ‘left–sright discrimination OR visuospatial attention OR body knowledge OR body space relation OR body schema OR self-rotation OR mental rotation AND PET’

Studies were only included if they satisfied the following search criteria: (i) peer reviewed publication, (ii) task-based or attention-based fMRI or PET study relating to one of the four neurocognitive domains impaired in the Gerstmann syndrome and (iii) reported standardized coordinate-based results in the Talairach or Montreal Neuroimaging Institute (MNI) coordinate space.

Overall, 102 articles met the criteria for inclusion in our study: 13 related to finger gnosis, 34 related to left–right discrimination, 27 related to writing and 28 for arithmetic.^18–120^ These studies ultimately included only fMRI studies as no PET studies were identified which also met all of our inclusion criteria.

### Machine-learning identification of relevant cortical regions from functionally activated regions

We used BrainMap GingerALE 2.3.6 to extract the relevant fMRI data from all 102 studies to create an ALE map.^121–123^ All of the Talairach coordinates identified during the literature review were converted to the MNI coordinate space using SPM Conversion in GingerALE. We subsequently performed a Single Study analysis using cluster-level interference in the MNI coordinate space (cluster level of 0.05, threshold permutations of 1000, uncorrected *P*-value of 0.001) for each of the functions separately. The ALE coordinate data was displayed on an MNI-normalized template brain using the Multi-image Analysis GUI (Mango; ric.uthscsa.edu/mango) 4.0.1.

A machine-learning approach was applied to determine relevant parcels based on the Human Connectome Project Multi-Modal Parcellation version 1.0 (HCP) atlas, which includes both cortical and subcortical parcels.^11^ This code was created by the above authors for the purpose of incorporating results from coordinate-based, meta-analyses into the HCP parcellation scheme and has been previously utilized by our team with great reproducibility.^124^ In brief, a sphere is placed over the MNI coordinate of each ALE cluster, with a radius of 15 mm. The sphere is projected onto the HCP parcellation schema which is also in MNI coordinates. The degree to which the sphere overlaps onto each HCP parcel (as a proportion of spherical volume) is calculated. The parcel with the most overlap is designated as the primary parcel, and the equivalent HCP parcel to that ALE cluster. A list of the included parcels was generated according to a requirement of at least 10% overlap, and ultimately included parcels were used to construct network models of each function. We provide a full list of the results of this process in the Supplementary Data.

There is great debate on the heterogenous classifications of Gerstmann’s syndrome that argues for 3, 4 or maybe more co-occurring neurologic deficits, which may explain the heterogenous battery of tests often employed in related studies and the subsequent rarity in its diagnosis.^5,6^ Furthermore, the presence of three or four Gerstmann symptoms has more lesion localizing value compared with two or less concomitant symptoms.^125^ Given that the primary goal of this study was to generate a cortical model which can provide clinically actionable anatomic information for clinical translations in this region, such as resective brain surgery, we sought to define our model based on a less strict definition that would not overestimate the precision of our statistical analyses. Therefore, we decided the Gerstmann Core was defined as the parcels that overlapped in at least three of four cognitive domains in order to capture the most underlying commonality in neuro-behavioural domains across the largest amount of data currently available in the literature.^5,125^ By using a strict cluster-level interference algorithm for our functional analyses, these three regions would provide a strong indication of the possible Gerstmann Core which likely best contributes to all four cognitive domains. Nonetheless, we also analyzed any regions which are shared amongst all *four* domains as well in agreement with some of the previous literature (See Supplementary Data for a full list of all ALE clusters and parcels per domain).^4^

### Structural network tractography

Complex experimental and computational data supports the hypothesis that *functionally* connected regions of a network tend to be *structurally* interconnected.^126–129^ Furthermore, the structural interconnectedness of a brain network places important constraints on the functional capacity of a network and neurologic functioning.^129^ Therefore, we utilized deterministic tractography based on publicly available neuroimaging data from the HCP database (http://humanconnectome.org, release Q3) to generate original data that could provide the basis of our cortical model. These methods have been reiteratively refined and replicated previously by our team with similar cortical regions and across the human cerebrum with great reproducibility between studies.^130–135^ Diffusion imaging with corresponding T_1_-weighted images from 51 healthy, unrelated subjects were analyzed during fibre tracking analysis. Subject IDs from this sample are presented in Supplementary Table 1.

The HCP data is minimally preprocessed as has been described previously.^136^ In brief, this Minimal Preprocessing Pipeline as described in Glasser *et al*^136^ includes basic preprocessing steps of (i) intensity normalization across runs, (ii) EPI distortion correction, (iii) eddy current and motion correction, (iv) gradient nonlinearity correction and calculation of gradient b-value/b-vector deviation, and (v) the registration of mean b0 values to native volume T_1_w space and bringing the diffusion data, gradient deviation and gradient directions into 1.25 mm structural space. Finally, (vi) it masks the data with the final brain mask on FreeSurfer segmentation.

All brains were registered to the Montreal Neurologic Institute (MNI) coordinate space.^137^ The imaging is warped to fit a standardized brain model comparison between subjects.^137^ Tractography was performed in DSI Studio (Carnegie Mellon, http://dsi-studio.labsolver.org) to better handle the possibility of ‘crossing fibres,’ which can be difficult to manage with DTI-based tractography in parietal regions.^138–140^ An ROI approach to initiate fibre tracking from a user-defined seed region was completed, namely the seeds being the specific parcels identified in our meta-analysis of the literature.^141^ A two-ROI approach was used to isolate tracts from seeded regions that selected from the overlapping activated cortical parcels identified in our ALE results.^142^ Voxels within each ROI were automatically traced with a maximum angular threshold of 45°. When a voxel was approached with no tract direction or a direction >45°, the tract was halted. The tracts were allowed to reach a maximum length of 800 mm before tractography was terminated. Exclusion ROIs had to be placed at times to exclude tracts that were obviously not involved in the white matter pathways of interest.

### Measuring connection strength

To quantify the strength of the connections identified within the four cognitive domains, the tracking parameters were modified such that the software would only count the total number of tracts between any two ROIs on a random seed count of 2.5 million. We then worked sequentially through the ROI pairs in the identified networks. We recorded the number of tracts between the regions for each of the subjects after deterministic fibre tractography was terminated under these conditions. The strengths of the white matter connections between the four cognitive networks, as well as within each network were computed by averaging the number of tracts between each ROI pair of the networks across all the subjects.

### Data availability

The data that support the findings of this study are available from the corresponding author upon reasonable request. Information about our ALE findings and structural results are presented in full in the Supplementary Data.

## Results

### Activation likelihood estimation regions and their corresponding parcellations

**Figure 1:**
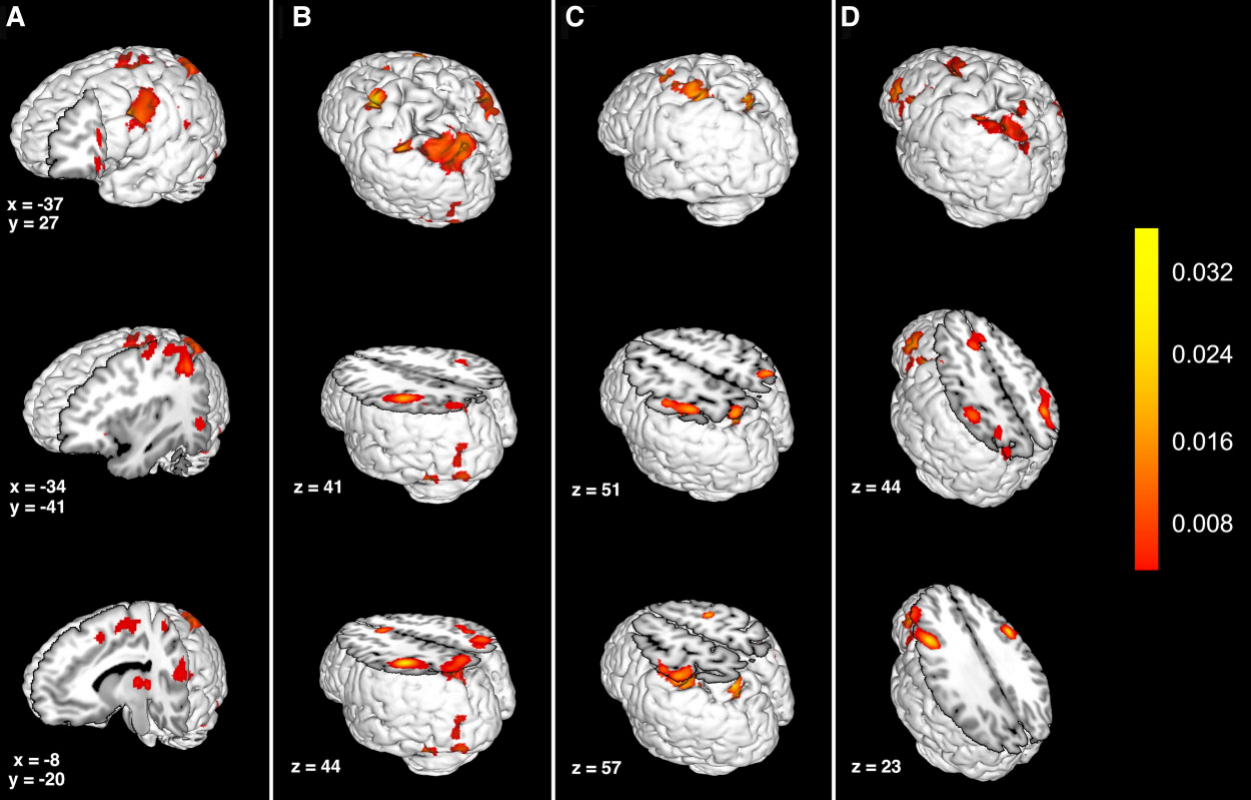
**The activation likelihood estimation for the neurocognitive domains involved in Gerstmann syndrome.** The activation likelihood estimation clusters of the task-based fMRI experiments that were included in our meta-analysis for (**A**) writing, (**B**) left–right discrimination, (**C**) finger agnosia and (**D**) arithmetic. The colour bar represents the ALE statistic, which increases in significance from bottom (red) to top (yellow).

**Figure 2:**
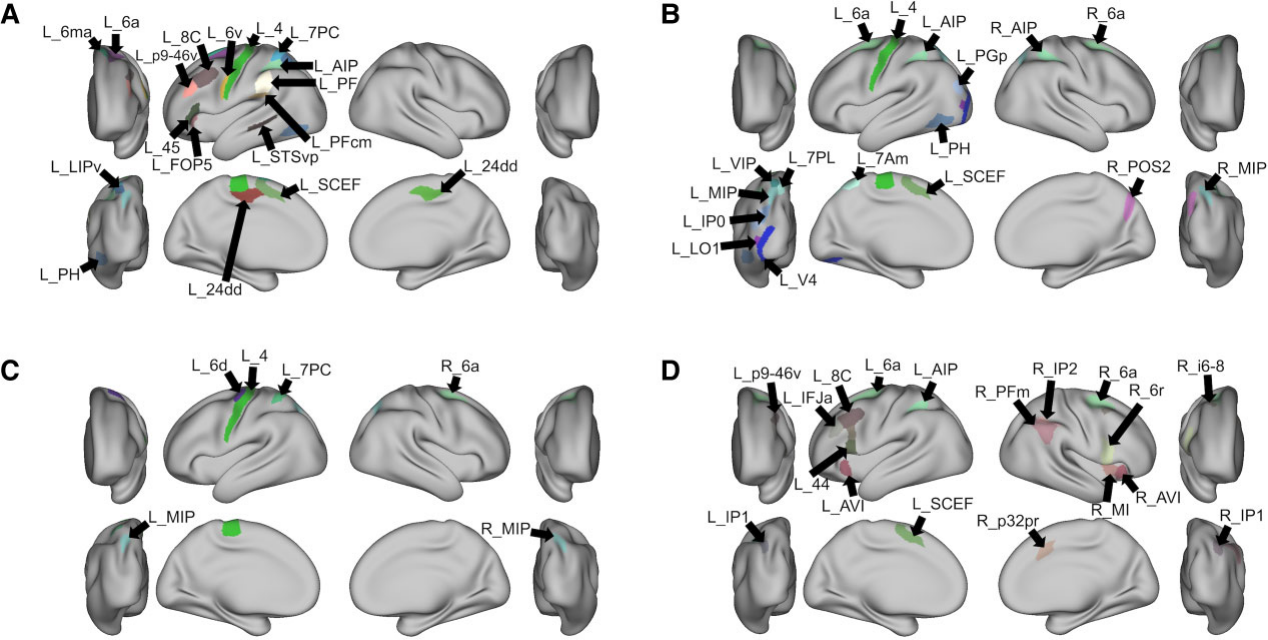
**The parcels with the largest percentage overlap with each activation likelihood estimation cluster.** Largest percentage overlap parcel for (**A**) writing, (**B**) left–right discrimination, (**C**) finger agnosia and (**D**) arithmetic. 24dd, Dorsal Area 24d; 6a, Area 6 Anterior; 6d, Dorsal Area 6; 6ma, Area 6m Anterior; 6r, Rostral Area 6; 6v, Ventral Area 6; 7Am, Medial Area 7A; 7PC, Area 7 postcentral; 7PL, Lateral Area 7P; 8C, Area 8C; AIP, Anterior Intraparietal Area; AVI, Anterior Ventral Insular Area; FOP5, Area Frontal Opercular 5; i6-8, Inferior 6-8 Transitional Area; IFJa, Area IFJa; IP0, Area Intraparietal 0; IP1, Area Intraparietal 1; IP2, Area Intraparietal 2; LIPv, Area Lateral Intraparietal Ventral; LO1, Area Lateral Occipital 1; MI, Middle Insular Area; MIP, medial Intraparietal Area; p32pr, Area p32 Prime; p9-46v, Area Posterior 9-46v; PF, Area PF Complex; PFcm, Area PFcm; PFm, Area PFm Complex; PGp, Area PGp; PH, area PH; POS2, Parieto-Occipital Sulcus Area 2; SCEF, Supplementary and Cingulate Eye Field; STSvp, superior temporal sulcus ventral posterior; VIP, Ventral Intraparietal Complex.

Figure 1 demonstrates the ALEs of the task-based fMRI experiments that were included in our meta-analysis, and Figure 2 demonstrates the parcel overlap for each ALE cluster.

### Writing

**Figure 3:**
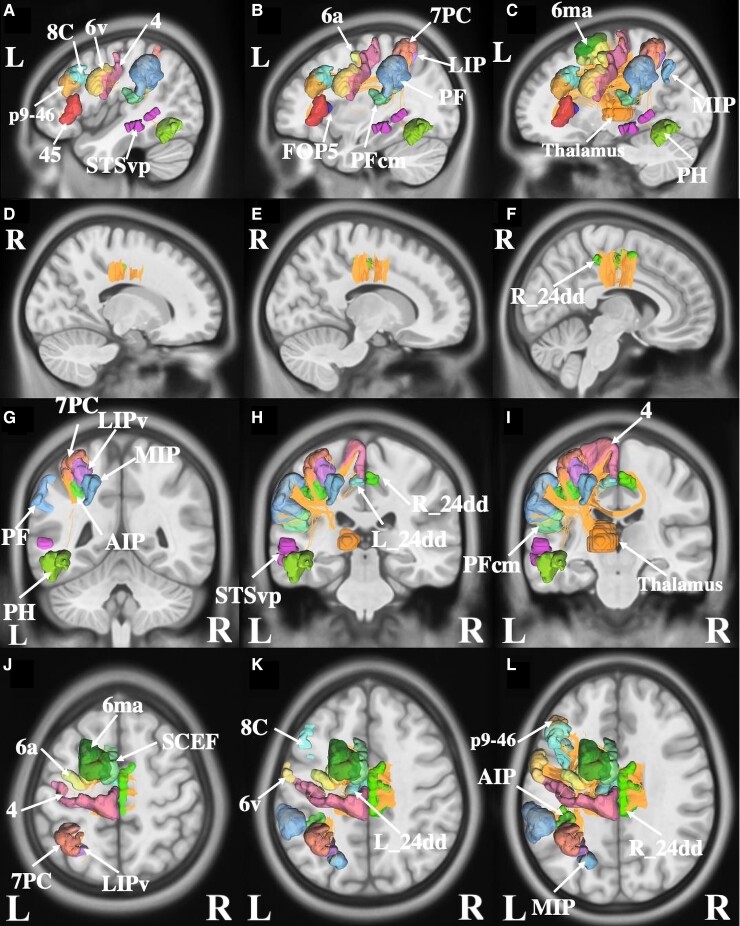
**Connectivity among parcels overlapping with the activation likelihood estimation for writing.** (**A–C**) sagittal sections, from lateral to medial, demonstrating the parcels in the left hemisphere that are involved in writing. (**D–F**) sagittal sections, from lateral to medial, demonstrating the parcels in the right hemisphere that are involved in writing. (**G–I**) coronal sections, from the posterior to anterior, highlighting the left and right-sided parcels and their tractography patterns. (J–L) axial sections, from superior to inferior, demonstrating the tractography and the anatomical relationships between parcels that contribute to writing.

We found 20 parcels that overlapped with the ALE for writing (Fig. 3). These included left-sided areas 7 postcentral (7PC), AIP (anterior intraparietal), lateral intraparietal, ventral, and medial intraparietal (MIP) in the intraparietal area, areas 6a (6 anterior), 6ma (6 medial anterior) and SCEF (supplementary and cingulate eye field) in the premotor regions. Other regions of interest included Area 4 in the motor strip, area PH, and superior temporal sulcus ventral posterior in the left temporal lobe, left-sided areas p9-46v (posterior 9-46 ventral) and 8C in the lateral frontal lobe, and left areas 6v (6 ventral), 45, thalamus, PFcm (parietal F, region CM), FOP 5 (frontal operculum 5), and bilateral 24dd (24 dorsal–dorsal).

### Left–right discrimination

**Figure 4:**
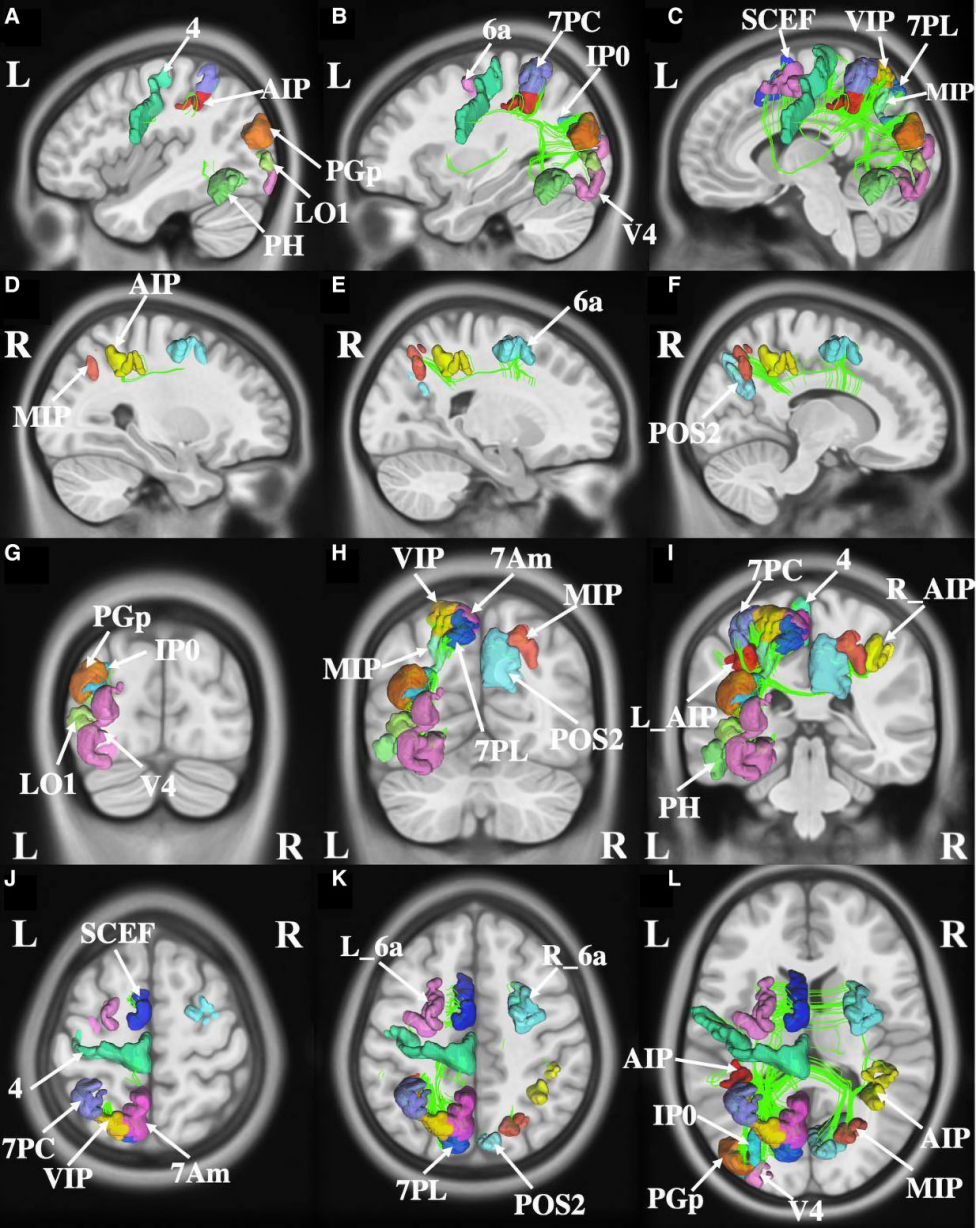
**Connectivity among parcels overlapping with the activation likelihood estimation for left–right discrimination.** (**A–C**) Sagittal sections, from lateral to medial, demonstrating the parcels and their tractography in the left hemisphere that are involved in the left–right discrimination. (**D–F**) Sagittal sections, from lateral to medial, demonstrating the parcels and their tractography in the right hemisphere that are involved in the left–right discrimination. (**G–I**) Coronal sections, from the posterior to anterior, highlighting the left and right-sided parcels and their tractography patterns. (**J–L**) Axial sections, from superior to inferior, demonstrating the tractography and the anatomical relationships between parcels that contributes to left–right discrimination.

Eighteen parcels overlapped with the ALE for left–right discrimination (Fig. 4). They included left-sided parcels 7PC, AIP, MIP in the intraparietal area, area 6a (6 anterior) and SCEF in the premotor region, Area 4 in the motor strip, areas 7PL (7 posterior–lateral), VIP (ventral intraparietal) and 7Am (7 anterior–medial) in the superior parietal areas, inferior parietal area PGp (parietal area G, posterior), lateral parietal area IP0 (intraparietal 0), PH in the temporal lobe and occipital areas V4 and LO1 (lateral occipital 1). In addition, right-sided MIP, POS2 (parieto-occipital sulcus 2), AIP and 6a were also overlapping the ALE.

### Finger agnosia

**Figure 5:**
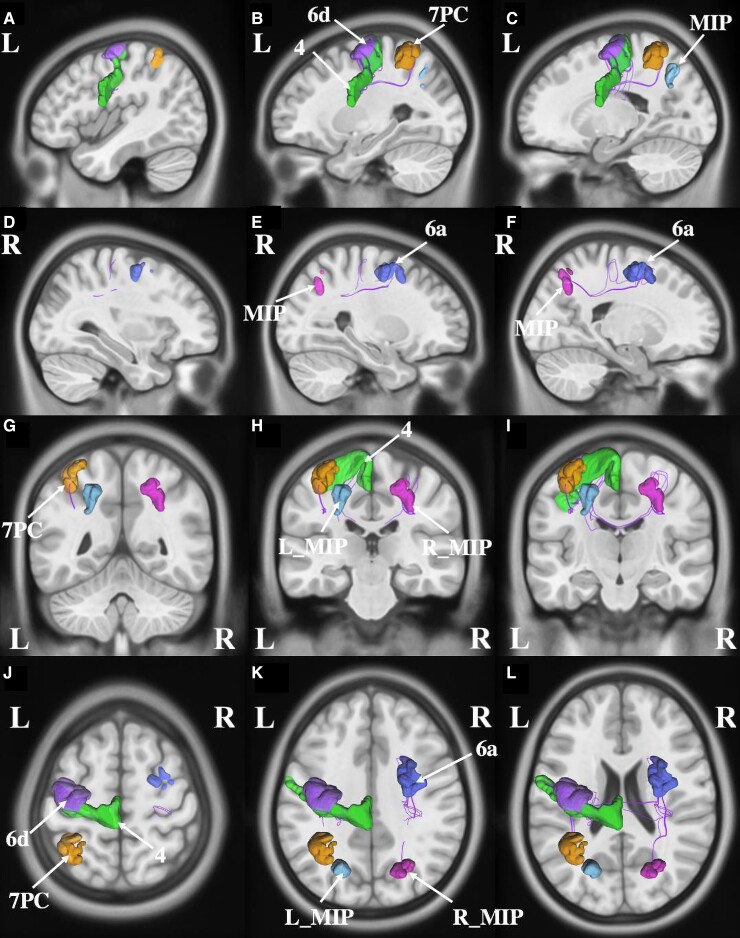
**Connectivity among parcels overlapping with the activation likelihood estimation for finger agnosia.** (**A–C**) Sagittal sections, from lateral to medial, demonstrating the parcels and their tractography in the left hemisphere that are involved in finger agnosia. (**D–F**) Sagittal sections, from lateral to medial, demonstrating the parcels and their tractography in the right hemisphere that are involved in finger agnosia. (**G–I**) Coronal sections, from the posterior, to anterior highlighting the left and right-sided parcels and their tractography patterns. (**J–L**) Axial sections, from superior to inferior, demonstrating the tractography and the anatomical relationships between parcels that contributes to finger agnosia.

Six parcels overlapped with the ALE for finger agnosia which included left-sided 7PC, MIP in the intraparietal area, Area 4 of the motor strip and 6d (6 dorsal) in the premotor region. Plus, right-sided MIP and 6a were also overlapping the ALE. These can be seen in Fig. 5.

### Arithmetic calculation

**Figure 6:**
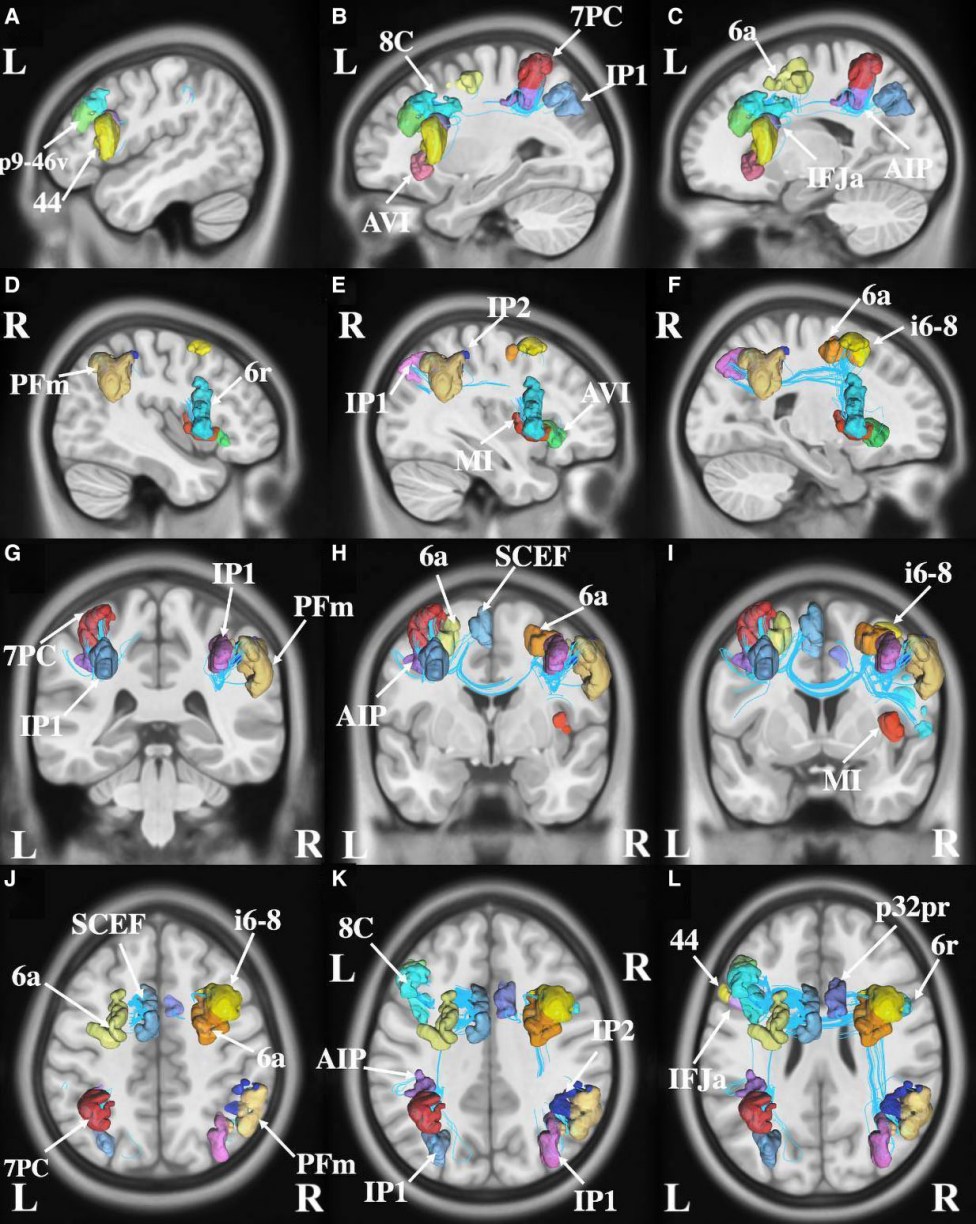
**Connectivity among parcels overlap with the activation likelihood estimation for arithmetic.** (**A–C**) sagittal sections, from lateral to medial, demonstrating the parcels in the left hemisphere that are involved in math. (**D–F**) sagittal sections, from lateral to medial, demonstrating the parcels in the right hemisphere that are involved in math. (**G–I**) coronal sections, from the posterior to anterior, highlighting the left and right-sided parcels and their tractography patterns. (**J–L**) axial sections, from superior to inferior, demonstrating the tractography and the anatomical relationships between parcels that contributes to math function.

Nineteen parcels overlapped with the ALE for the arithmetic core including left-sided 7PC, AIP and IP1 (intraparietal 1) in the lateral parietal lobe, 6a and SCEF in the premotor region, p9-46v, 44 and 8C in the lateral frontal lobe, inferior frontal junction area in the inferior frontal lobe, anterior ventral insula (AVI) in the insula, as seen in Fig. 6. In addition, right-sided parcels AVI, middle insula, 6 rostral, p32pr (posterior 32 prime), IP1, IP2 (intraparietal 2), PFm (parietal F, part m), 6a and i6-8 (inferior 6-8) were also found.

### Gerstmann core network

**Figure 7:**
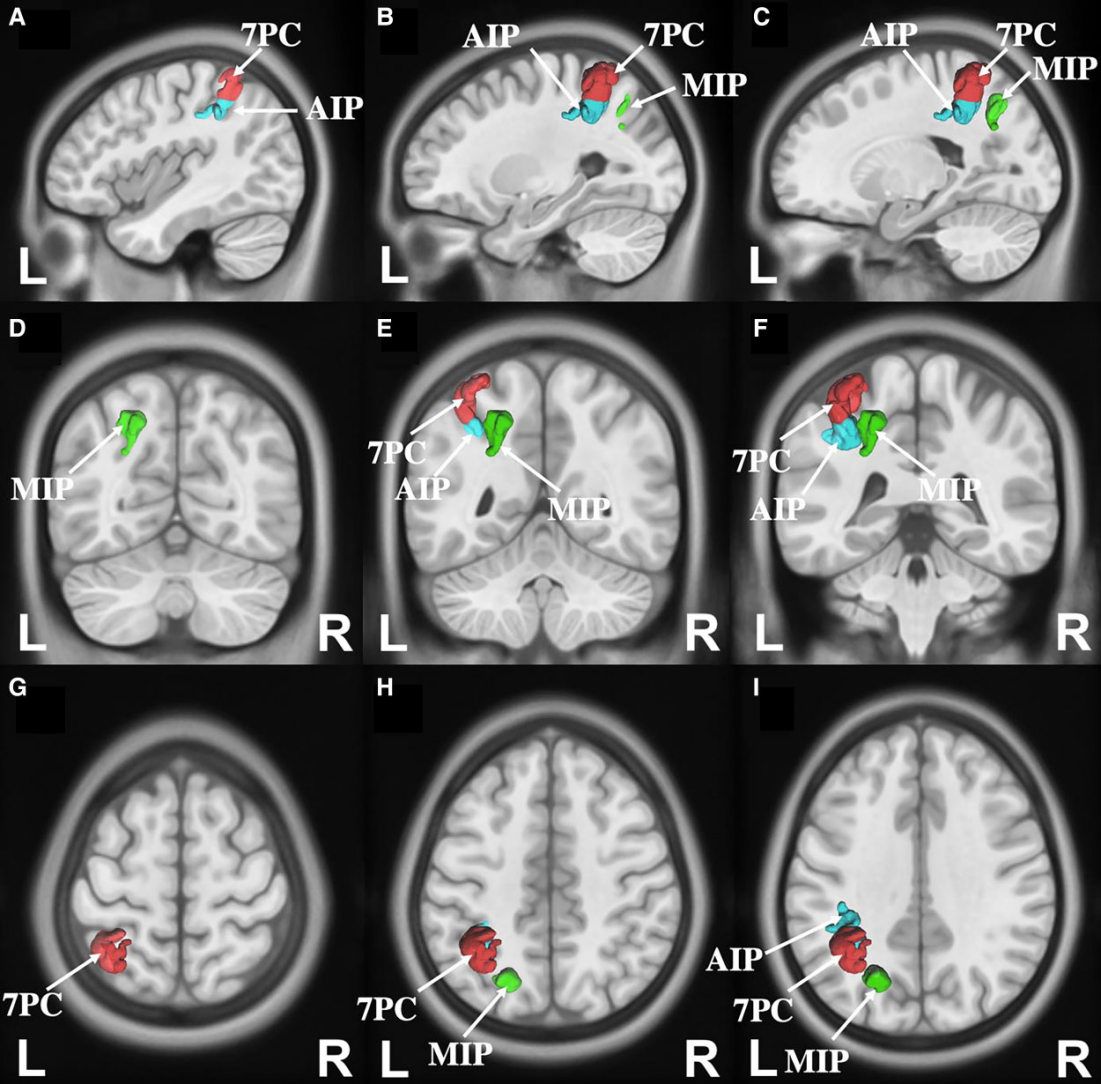
**Parcels that represent the core of the Gerstmann’s syndrome.** 7PC (red), MIP (green) and AIP (blue) in the left hemisphere. (**A–C**) Sagittal sections, from lateral to medial (**D–F**) coronal sections, from the posterior to anterior. (**G–I**) Axial sections, from superior to inferior.

**Figure 8:**
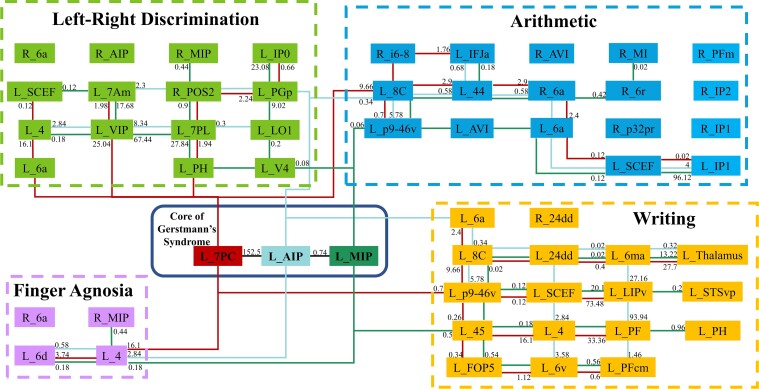
**Simplified schematic of the white matter connections identified between individual parcels and the core of the Gerstmann’s syndrome.** L, left. R, right. Resultant tract volumes are listed for each parcel which is connected directly on the diagram. Of note is the connecting lines do not show a relationship between all parcels they connect, but rather the three parcels in the Gerstmann Core with all other parcels, and a true connection is only there if there is an associated number. The number associated with each connection represents the average number of streamlines between given two regions on tractography across all subjects.

The ROIs to be included in the core of the Gerstmann’s syndrome were arrived at by identifying the parcels where the ALEs overlapped in at least three or all of the four cognitive domains. These were the left 7PC, left MIP and left AIP, which are all present in the intraparietal sulcus. If considering the definitions provided by some other authors in the literature, evidence for a single shared functional regions was also identified across all four cognitive domains, namely area left 7PC. These results are represented in Fig. 7 and a simplified schematic diagram that highlights the white matter connections between the core of Gerstmann’s syndrome and other parcels is also shown in Fig. 8.

Since writing and mathematical calculations require an intact language network, the involved parcels were exclusively left sided. Left–right discrimination and finger gnosis, on the other hand did involve right-sided parcels and hence were bilateral since these tasks are not strictly dependent on the language network. Areas that were in the motor or premotor regions were not included in the Gerstmann Core as tasks that are required to activate the relevant functions often simultaneously involve the sensorimotor areas and therefore may decrease the specificity of our model.^5^

### Structural connectivity of the four neurocognitive domains and their relationship to gerstmann core

Deterministic tractography determined the structural connectivity between the different networks and their connections to the Gerstmann Core. The 51 healthy, unrelated subjects from the HCP database which were included in these analyses demonstrated a mean age of 28.4 years and consisted of slightly more females (51%) than males (49%). The simplified structural results are shown in a schematic wire diagram Fig. 8. In Supplementary Material Table 1, we show the average number of tracts identified between the core parcels and between each domain network according to *intra*- and *inter*-network connections. We also provide the average number of connections between each pair of parcels across each subject in the Supplementary Material Table 2.

The cortical regions included in the connections can be categorized into clusters based on the large-scale brain networks they form a part of. We identified a multiple demand (MD) network cluster (left MIP, left p9-46v, left IP1, right MIP, and right AIP), a sensorimotor cluster (left 4, left 6v, left 6d, left 6a, left 24dd, right 24dd, and right 6a) and dorsal attention network (DAN) cluster (left 7PC, left IPv, left PF, left PH, left 7PL, left VIP, and left 7Am). The remaining cortical regions form a part of the language, salience, visual and central executive networks.

Numerous local, short-association white matter bundles were found linking the Gerstmann Core. These intraparietal fibres are likely part of the larger superior longitudinal fasciculus (SLF), which links many of the Gerstmann Core regions with the additional extra-core parcels discussed above.^143^ The SLF projects between the frontal, parietal and occipital areas of the networks, as it courses within the subcortical white matter around the Sylvian fissure (Fig. 3).

## Discussion

We sought to examine the available literature for evidence of a network of cortical regions which may underlie Gerstmann’s syndrome, which includes unique deficits of agraphia, acalculia, left–right discrimination and finger agnosia. Through a meta-analysis of 102 task-based fMRI studies in combination with probabilistic tractography on 51 HCP subjects, we provide a novel connectivity model of the neural correlates underlying the diverse functions which are impaired in Gerstmann’s syndrome. Namely, three specific parcels were identified which converge in the left anteromedial portion of the intraparietal sulcus and are structurally connected through numerous short-association bundles, together in what we deem the ‘Gerstman Core’ network. These results align well with previous work demonstrated in both lesion study^10^ and in healthy subjects^9^ which suggest a possible disconnection origin of Gerstmann’s syndrome along the intraparietal sulcus given their connectivity, however with the additional benefits provided by our more powered meta-analytic methodology and further tractographic data on the structural connectivity of this region. Furthermore, while not possible in earlier studies, we discuss the localization of identified regions to more recently characterized, large-scale brain networks involved in higher-order functions so that we may gain additional unique insight and localizational value into Gerstman’s syndrome based on a network perspective.

Previous results have been limited in their level of anatomic granularity which is necessary for improved reproducibility between studies and to guide effective clinical decision making in clinical neuroscience, such as during resective brain surgery which inevitably involves making difficult surgical cuts and relies on fine precision. Therefore, we incorporate our results into an anatomically specific parcellation scheme outlined by the HCP. While a pure form of Gerstmann’s syndrome is extremely unlikely, many of these cortical regions are commonly encountered in clinical settings and therefore our results may serve as an empiric basis for future study on these domains and more effective clinical translation in the context of Gerstmann’s syndrome.

### The precise localization of the Gerstmann core in the intraparietal sulcus

We defined the Gerstmann Core as the region in the cortex where the parcels are involved in at least three of the four cognitive domains implicated in Gerstmann’s tetrad. This definition was chosen to most accurately identify the relevant neural correlates of the functions which are disrupted in Gerstmann’s syndrome for effective clinical translation without overestimating the precision of our analyses with a stricter definition. Furthermore, there has been previous discussion on the predictive benefits of when at least three or four Gerstmann symptoms arise together and debate on the required amount of co-occurrences of symptoms for a true diagnosis of Gerstmann’s syndrome.^5,6,125,144^ Such limitations have likely contributed to the rarity of this diagnosis and also a lack of clinically actionable anatomic information, despite many of Gerstmann’s symptoms being commonly identified in isolation or slightly different combinations in the clinical context, such as also with aphasia or apraxia.^125^ Here, our results suggest a statistically likely network of neural correlates for the functions which are impaired in Gerstmann’s syndrome, including the parcels anterior intraparietal area (AIP), area 7PC and MIP, specifically in the left hemisphere. However, if we were to extend our threshold to similar strict requirements as some previous authors,^4,9^ our results still identified plausible evidence for at least one specific parcel possibly underlying all four cognitive functions, specifically area 7PC which is distinct from its adjacent cortical regions according to cytoarchitectonic analyses.^145^

Anatomically, area 7PC is located in the anterior-inferior portion of the superior parietal lobe and areas MIP and AIP in the superior bank of the intraparietal sulcus at its most posterior and anterior aspects, respectively.^11,146^ While we elucidate the functional relevance of each region further in the Supplementary Material Table 3, multiple lines of previous work have suggested key roles of areas AIP, MIP and 7PC in complex processing related to arithmetic abilities, fine finger representations, left–right orientation and handwriting.^21,50,89,90,124,147^ There has been great debate about the localization of damage that disrupt of all of these functions together.^2,5,6^ One of the best accounts of a pure form of acquired Gerstmann’s syndrome has been reported following a focal ischaemic lesion in the anterior intraparietal sulcus region,^10^ which overlaps well with network location in the current results. Importantly, this lesion was presumed to be in a subcortical pathway, causing increased speculation about a possible disconnection origin of Gerstmann’s syndrome.^9^

In a combined functional and structural study on five healthy subjects, Rusconi *et al*.^9^ demonstrated that functionally activated cortical regions corresponding to the functions underlying Gerstmann’s tetrad are all *structurally* connected through shared, short-range white matter fibre bundles in the intraparietal sulcus, and that this is further supported by lesion simulated approaches. Results from the present study provide the first support for this previous work, despite our different methodological approaches which did not utilize high-resolution single subject imaging as in Rusconi *et al*., but rather utilized primarily larger amounts of low-resolution data from fMRI group studies and then secondary probabilistic methods with ALE. Furthermore, while the intraparietal sulcus is a heterogenous region consisting of numerous functional regions, our results provide strong evidence that specifically area 7PC in the left hemisphere is involved in all the functions which are impaired in Gerstmann’s tetrad, as well as areas AIP and MIP also likely being involved albeit with less evidence in the current study.

### The benefits of a more precise and connectomic understanding of the Gerstmann core

One of the major benefits of the current study is the incorporation of results into a previously established and highly granular parcellation scheme published under the HCP.^11^ While previous studies have noted regions of the intraparietal sulcus or dominant parietal lobe, such as the angular gyrus, may be most responsible for Gerstmann’s syndrome, they have lacked the anatomic specificity necessary to convey specifically which aspect of which gyri or sulci is of clinical interest or must be preserved during surgery.^3,4,140^ The use of a surface-based parcellation scheme provides a more effective means for a data-driven analysis approach with better reproducibility and hypothesis refinement between studies.^148^ Importantly, regulated medical devices are now capable of incorporating this highly granular anatomic information into the operating room to guide clinical neuroscience decisions.^13^ Previously, due to a lack of information surrounding both the precise localization and connectivity of the likely anatomic substrates underlying the functions related to Gerstmann’s syndrome, it has been difficult for neurosurgeons to preserve many of these functions given that operating in the dominant parietal lobe inevitably requires some cuts to be made.^149,150^ However, more anatomically specific results in a parcellation scheme can demonstrate with greater precision exactly which cortical regions or connections along the intraparietal sulcus must be preserved to avoid inducing many of the symptoms involved in Gerstmann’s tetrad, if not all of them together.

Recent improvements in neuroimaging capabilities and the use of more specific cortical parcellation atlases have greatly improved our understanding of the architecture of large-scale brain networks throughout the brain connectome. Large-scale brain networks include spatially distant but highly synchronized brain regions and are known to subserve complex human functioning and behaviour, but have not been discussed in previous studies on Gerstmann syndrome given their relative nascent characterization.^151^ Therefore, our incorporation of results into an anatomically specific parcellation scheme in the context of large-scale brain networks provides significant insight and localizational value into this disease that has been previously limited.^11,14^ Namely, regions in the Gerstmann Core have been previously demonstrated by others and our team to be involved in the DAN^152^ and MD network (Supplementary Material Table 3).^153^ Both of these networks have been implicated in a variety of cognitive processes such as in the attentional control required for mathematic processes or writing, but particularly these functions are subserved through both intra-network connections around the intraparietal sulcus as well as extra-network interactions with other higher-order brain networks.^12,154^ Each domain included a number of parcels which are known to affiliate with higher-order networks like the sensorimotor, salience, language, and central executive networks. Thus, while previous localizationist views often suggest neurologic deficits can be localized to a single region, such as a speech deficit following surgical injury to Broca’s area, Gerstmann’s symptoms may likely be better localized to dysfunction in a number of large-scale brain networks which dynamically interact to carry out many higher-order functions.^14^ Our connectivity model of these likely regions and their white matter connections details this anatomy in a way which can offer prognostic information for neurosurgeons during intra-axial, resective brain surgery such that we can make more informed surgical decisions while causing fewer deficits according to patient functional goals.^12,126^

### Limitations

While the present study outlines a structural and functional model of a plausible Gerstmann Core and lends supports to previous cortical models, this study is not without its limitations. Importantly, coordinate-based ALE analyses allow the procuring of foci reported from numerous different experiments which can improve study power and the ability to generate hypotheses for further discussion.^130^ However, this method is limited by the quality of the reported data in the literature and therefore demonstrates possible selection and publication biases.^155,156^ Furthermore, *connectivity*-based neuroimaging studies can often overcome limitations faced by *focal* lesion analyses which do not truly consider the whole brain network.^129^ However, the current study only included healthy subjects and therefore the degree to which our results may be influenced by pathogenic mechanisms of injury or dysfunction remains unknown, and thus our subsequent inferences about how this model applies to patients with Gerstmann’s syndrome are purely speculative. It may be advantageous for meta-analyses of the available literature which also include lesion-based studies to better derive clinical meaning to these results.^157^

Nonetheless, these limitations are commensurate with the value provided by the present work according to its study goals. Our connectivity results suggest possible underlying neural correlates which align well with previous reports and studies of Gerstmann’s syndrome,^9,10^ however with additional power from meta-analytic methodology as well as in a finer anatomic nomenclature necessary for effective clinical translation and hypothesis comparison in future work. Furthermore, with additional information provided by our connectivity analyses, this work may serve as an empiric basis for future study on Gerstmann’s syndrome as a result of white matter disconnection from a network perspective.

## Conclusions

We present an anatomically specific connectivity model of the parcels involved in the neurocognitive domains that are affected by the Gerstmann’s syndrome, which specifically localizes in the anteromedial portion of the intraparietal sulcus. These results provide convergent support for previous lesion and healthy structural–ssfunctional analyses provided in the literature. Together, it is likely that specifically area 7PC and areas AIP and MIP of the anterior intraparietal sulcus are the key cortical areas underlying a plethora of cognitive impairments following damage or disconnection in the dominant parietal lobe. The present study highlights for future work the feasibility in utilizing similar meta-analytic and combined structural–functional analyses to investigate other rare, multi-functional disorders following possible disconnection and from a connectomic standpoint.

## Supplementary Material

fcac140_Supplementary_Data

## Abbreviations

24dd =: area 24 dorsal–dorsal
6a =: area 6 anterior
6d =: area 6 dorsal
6ma =: area 6 medial anterior
6r =: area 6 rostral
6v =: area 6 ventral
7Am =: medial area 7A
7PL =: lateral area 7P
ALE =: activation likelihood estimation
AIP =: anterior intraparietal area
AVI =: anterior ventral insular area
DAN =: dorsal attention network
DSI =: diffusion spectrum imaging
fMRI =: functional MRI
FOP5 =: area frontal opercular 5
HCP =: Human Connectome Project
i6-8 =: inferior 6-8 transitional area
IFJa =: inferior frontal junction area
IP0 =: area intraparietal 0
IP1 =: area intraparietal 1
IP2 =: area intraparietal 2
LIPv =: area lateral intraparietal ventral
LO1 =: area lateral occipital 1
MD =: multiple demand
MI =: middle insular area
MIP =: medial intraparietal sulcus
p32pr =: area p32 prime
p9-46v =: area posterior 9-46v
PFm =: area PFm complex
POS2 =: parieto-occipital sulcus area 2
ROI =: region of interest
SCEF =: supplementary and cingulate eye field
SLF =: superior longitudinal fasciculus
STSvp =: area superior temporal sulcus ventral posterior
VIP =: ventral intraparietal complex.
