# Supplementary material for: A connectivity model of the anatomic substrates underlying Gerstmann syndrome
Source: Brain Commun. 2022 May 27;4(3):fcac140. doi: 10.1093/braincomms/fcac140 (PMC9189613; doi:10.1093/braincomms/fcac140)
Supplement: fcac140_Supplementary_Data [file fcac140_supplementary_data.zip › Supplementary_Material.docx]

*Supplementary Material*: A connectivity model of the anatomic substrates underlying Gerstmann syndrome

Qazi S. Shahab^1^, Isabella M. Young^2^, Nicholas B. Dadario^3^, Onur Tanglay^2^, Peter J. Nicholas^2^, Yueh-Hsin Lin^4^, R. Dineth Fonseka^4^, Jacky T. Yeung^4^, Michael Y. Bai^4^, Charles Teo^4^, Stephane Doyen^2^ and Michael E. Sughrue^2,4^

**Author affiliations:**

1 School of Medicine, University of New South Wales, 2052, Sydney, Australia

2 Omniscient Neurotechnology, Sydney, 2000, Australia

3 Rutgers Robert Wood Johnson Medical School, New Brunswick, New Jersey 08901, United States of America

4 Centre for Minimally Invasive Neurosurgery, Prince of Wales Private Hospital, Randwick, 2031, Australia

| **Supplementary Table 1. Tractography Subjects.** List of 51 healthy, unrelated subjects from the Human Connectome Project (HCP) database (http://humanconnectome.org, release Q3) in which diffusion imaging with corresponding T1‐weighted images was utilized for fiber tracking analyses. | | | | |
| --- | --- | --- | --- | --- |
| 100206 | 100307 | 100408 | 100610 | 103414 |
| 105115 | 110411 | 111312 | 113619 | 115320 |
| 116423 | 116524 | 117122 | 117930 | 118730 |
| 118932 | 123925 | 144832 | 148335 | 148840 |
| 151526 | 160123 | 163432 | 178950 | 185038 |
| 188347 | 192540 | 198653 | 209329 | 212318 |
| 214726 | 295146 | 307127 | 348545 | 355239 |
| 366446 | 385450 | 448347 | 520228 | 580347 |
| 589567 | 592455 | 657659 | 677968 | 748258 |
| 756055 | 779370 | 865363 | 872764 | 978578 |
| 995174 |  |  |  |  |

**Supplementary Table 2**. The average number of white matter connections according to diffusion-spectrum imaging (DSI)-based tractographic analyses is presented based on parcellations in each cognitive domain as well as the parcellations only in the Gerstmann’s core (L_7PC, L_AIP, L_MIP).

Abbreviations, 7PC (area 7 postcentral), AIP (anterior intraparietal area), and MIP (medial intraparietal sulcus)

| *Intra*- and *Inter*- Network Connections for Each Domain | | | | |
| --- | --- | --- | --- | --- |
|  | **Writing** | **Left-Right Discrimination** | **Finger Agnosia** | **Arithmetic** |
| **Writing** | 371.645 | 160.881 | 97.818 | 190.202 |
| **Left-Right Discrimination** |  | 100.769 | 61.413 | 96.763 |
| **Finger Agnosia** |  |  | 69.647 | 204.227 |
| **Arithmetic** |  |  |  | 192.257 |
|  |  |  |  |  |
| Network Connections for the Gerstmann Core | | | | |
|  | **Writing** | **Left-Right Discrimination** | **Finger Agnosia** | **Arithmetic** |
| **L_7PC** | 330.74 | 214.94 | 31.8 | 174.04 |
| **L_AIP** | 292.26 | 167.02 | 156.66 | 159.9 |
| **L_MIP** | 49.18 | 160.64 | 12.76 | 109.64 |

| Table 3. Gerstmann Core Network | | | | | | | |
| --- | --- | --- | --- | --- | --- | --- | --- |
| **Parcellation** | **Coordinates in MNI Space** | | | **Cluster Size** | **Relative cortical location** | **Brain Network**  **Affiliation** | **Speculative Function** |
|  | ***x*** | ***y*** | ***z*** |  |  |  |  |
| **Area 7 Postcentral (7PC)** | -27 | -62 | 43 | 3 mm^3^ | Anterior, inferior superior parietal lobule | Dorsal Attention Network | Visual and somatosensory processing; guiding and executing visuomotor behavior in space; imagination |
| **Anterior Intraparietal (AIP)** | -38 | -44 | 43 | 4 mm^3^ | Anterior aspect of intraparietal sulcus | Multiple Demand | Object recognition, processing, and grasping; visuospatial processing |
| **Medial Intraparietal (MIP)** | -27 | -59 | 41 | 2 mm^3^ | Posterior aspect of superior bank of intraparietal sulcus | Multiple Demand | Manipulation of objects in space through arm adjustment and error correction after processing of relevant proprioceptive and visual signals |

**MIP Area**

Neurons in the MIP area are modulated by visual and somatosensory information, with the majority of the short association bundles projecting inferiorly to IP1, a part of the arithmetic core.^1^ This area is important for the transformation of visual cues into motor action for precise movements as well as for the correction of movement error.^2^ Area MIP has been implicated in a variety of tasks such as, imitation of hand movements, left right discrimination and handwriting.^3-5^

**AIP Area**

Neurons in the AIP area facilitate understanding of orientation in space. AIP receives input from the ventral and dorsal visual streams which plays a critical role in orienting the hand for grasping objects.^1^ It has been demonstrated to be involved in discriminating between left and right hands.^6^ Furthermore, AIP has functional connectivity with other parcellations in the intraparietal sulcus which include IP0 and IP1. These parcellations in the anterolateral bank of the intraparietal sulcus are involved in complex processing related to arithmetic activities and finger representations.^1, 7^ AIP also appears to be involved in processes related to fine finger movements and lexical and written tasks, with increased activations surrounding the left perisylvian fissure.^8, 9^

**Area 7PC**

Area 7PC is involved in the observation of space and visual motion.^1^ In particular, the left hemispheric portion of 7PC is associated with imagination, a technique which has been utilised in studies aimed at identifying the neural substrates of left-right orientation.^10-13^ Additionally, this area forms a part of the dorsal attention network (DAN), which has been demonstrated to be important in one’s arithmetic ability, as it allows for an increased ability to suppress distracting information to flexibly switch one’s attention between tasks.^14^

**Supplementary References**

1. Baker CM, Burks JD, Briggs RG, et al. A Connectomic Atlas of the Human Cerebrum-Chapter 7: The Lateral Parietal Lobe. *Oper Neurosurg (Hagerstown)*. 12 2018;15(suppl_1):S295-S349. doi:10.1093/ons/opy261

2. Baker CM, Burks JD, Briggs RG, et al. A Connectomic Atlas of the Human Cerebrum-Chapter 8: The Posterior Cingulate Cortex, Medial Parietal Lobe, and Parieto-Occipital Sulcus. *Oper Neurosurg (Hagerstown)*. 12 2018;15(suppl_1):S350-S371. doi:10.1093/ons/opy262

3. Tanaka S, Inui T. Cortical involvement for action imitation of hand/arm postures versus finger configurations: an fMRI study. *NeuroReport*. 2002/09 2002;13(13):1599-1602. doi:10.1097/00001756-200209160-00005

4. Roux F-E, Dufor O, Giussani C, et al. The graphemic/motor frontal area Exner's area revisited. *Annals of Neurology*. 2009/07/20 2009;66(4):537-545. doi:10.1002/ana.21804

5. Roux F-E, Durand J-B, Réhault E, Planton S, Draper L, Démonet J-F. The neural basis for writing from dictation in the temporoparietal cortex. *Cortex*. 2014/01 2014;50:64-75. doi:10.1016/j.cortex.2013.09.012

6. Bonda E, Frey S, Petrides M. Evidence for a dorso-medial parietal system involved in mental transformations of the body. *Journal of Neurophysiology*. 1996/09/01 1996;76(3):2042-2048. doi:10.1152/jn.1996.76.3.2042

7. Andres M, Michaux N, Pesenti M. Common substrate for mental arithmetic and finger representation in the parietal cortex. *Neuroimage*. Sep 2012;62(3):1520-8. doi:10.1016/j.neuroimage.2012.05.047

8. Catalan M. The functional neuroanatomy of simple and complex sequential finger movements: a PET study. *Brain*. 1998/02/01 1998;121(2):253-264. doi:10.1093/brain/121.2.253

9. DeMarco AT, Wilson SM, Rising K, Rapcsak SZ, Beeson PM. Neural substrates of sublexical processing for spelling. *Brain and language*. 2017;164:118-128. doi:10.1016/j.bandl.2016.10.001

10. Creem SH, Downs TH, Wraga M, Harrington GS, Proffitt DR, Downs JH. An fMRI study of imagined self-rotation. *Cognitive, Affective, & Behavioral Neuroscience*. 2001/09 2001;1(3):239-249. doi:10.3758/cabn.1.3.239

11. Jordan K, Heinze HJ, Lutz K, Kanowski M, Jäncke L. Cortical Activations during the Mental Rotation of Different Visual Objects. *NeuroImage*. 2001/01 2001;13(1):143-152. doi:10.1006/nimg.2000.0677

12. Lamm C, Windischberger C, Leodolter U, Moser E, Bauer H. Evidence for Premotor Cortex Activity during Dynamic Visuospatial Imagery from Single-Trial Functional Magnetic Resonance Imaging and Event-Related Slow Cortical Potentials. *NeuroImage*. 2001/08 2001;14(2):268-283. doi:10.1006/nimg.2001.0850

13. Chaminade T, Meltzoff AN, Decety J. An fMRI study of imitation: action representation and body schema. *Neuropsychologia*. 2005;43(1):115-127. doi:10.1016/j.neuropsychologia.2004.04.026

14. Cragg L, Gilmore C. Skills underlying mathematics: The role of executive function in the development of mathematics proficiency. Trends in Neuroscience and Education: Elsevier; 2014. p. 63-68.
